# The Comparative Effect of Morphine on Proliferation of Cancer Cell Lines Originating from Different Organs: An In Vitro Study

**DOI:** 10.3390/ph17121656

**Published:** 2024-12-09

**Authors:** Lydia Whitham, Mahdi Sheikh, Markus W. Hollmann, Marie-Odile Parat

**Affiliations:** 1 School of Pharmacy, University of Queensland, Brisbane 4072, Australia; l.whitham@student.uq.edu.au; 2 Genomic Epidemiology Branch, International Agency for Research on Cancer (IARC-WHO), 69007 Lyon, France; sheikhm@iarc.who.int; 3 Department of Anaesthesiology, Amsterdam University Medical Center, 1081 Amsterdam, The Netherlands; m.w.hollmann@amsterdamumc.nl

**Keywords:** morphine, proliferation, cancer cell lines

## Abstract

**Background/Objectives**: Opium consumption was recently classified by the International Agency for Research on Cancer (IARC) monograph as carcinogenic to humans based on strong evidence for cancers of the larynx, lung, and urinary bladder, and limited evidence for cancers of the oesophagus, stomach, pancreas, and pharynx. This poses the question of a potential pro-cancer effect of pharmaceutical opioid analgesics. In vitro studies employing a variety of experimental conditions suggest that opioid alkaloids have proliferative or antiproliferative effects. We set out to reconcile this discrepancy and explore the hypothesis that opioids promote cancer cell proliferation in an organ-dependent fashion. **Methods**: Using strictly controlled conditions, we tested the effect of morphine on the proliferation of a series of human cancer cell lines isolated from organs where cancer risk was linked causally to opium consumption in human studies (i.e., lung, bladder, and larynx), or control organs where no link between cancer risk and opium consumption has been reported in human studies (i.e., breast, colon, prostate). **Results**: Our results showed a minimal effect on proliferation on any cell line and no trend supporting an organ-specific effect of morphine. **Conclusions**: This argues against a direct effect of opioids on tumour cell proliferation to support their organ-specific effect.

## 1. Introduction

Recent studies report a pain prevalence of 44.5% in cancer patients [1], which can be caused by direct tumour involvement or adverse effects from surgery or chemotherapeutic treatment [2]. Cancer-related pain is commonly managed by a step-up approach including low-dose opioids in the initial stages and up-titration with increasing pain [3]. Opioids may therefore be used for a long duration in cancer patients. Although no longer considered to be a first-line option [4], opioids are also commonly used in management of non-cancer chronic pain, with an estimated 20–50% of the population experiencing chronic pain and an estimated 30% of this group using opioids to manage it [5]. In this setting, opioids may also be used for a long duration and in high doses due to the development of tolerance, with 18.4% of chronic pain patients reportedly treated with a strong opioid and 24.1% being treated with strong combination opioids [5].

It is important to determine whether opioids increase the risk of developing cancer in chronic pain patients or promote the growth of existing tumours in cancer patients. Studies have demonstrated that opioids can promote or prevent cancer using in vitro (proliferation [6], migration [7], invasion [8], etc.) assays and in vivo (tumour growth [9], burden [10], angiogenesis [11], metastasis [12], etc.) models. To explain this discrepancy, it has been suggested that the effect of opioids on proliferation may be drug-dependant based on varying affinities for receptors and downstream effects between different opioid molecules [3,13]. Another factor proposed to underlie the variation in opioid effects on tumour growth and metastasis is the dose (in vivo) or concentration (in vitro) of an opioid used, as multiple experimental conditions included doses/concentrations either higher or lower than clinically relevant [14,15,16]. Lastly, several studies have evaluated whether the effect of opioids on tumour growth is receptor-dependant and involves traditional opioid receptors [17,18,19] or non-opioid receptors [6,17,20,21,22,23,24].

Interestingly, a new explanation for the apparent discrepancy in the literature on the effect of opioids on cancer has recently emerged, with the possibility that opioid alkaloids may promote cancer development in some but not all organs. The International Agency for Research on Cancer (IARC) of the World Health Organisation (WHO) recently listed opium consumption as carcinogenic to humans, based on strong evidence for cancers of the larynx, lung, and bladder [25]. The IARC monograph reported that smoking or ingesting opium was associated with a more than twofold increased risk for developing cancers of the larynx, lung, and bladder [25]. The review also found limited evidence suggesting that opium consumption increases the risk for cancers of the oesophagus, stomach, pancreas, and pharynx, with no evidence of an effect on cancer risk in other organs. Of note, emerging evidence indicates a similar effect for pharmaceutical opioid molecules [26,27].

This novel finding poses the question of the mechanism by which opioids would differently affect tumour growth in different organs. One possibility is that the response to opioids of the cancer cells themselves differs between organs. Morphine is the most abundant alkaloid present in opium (~12% of raw opium weight) [28] and is widely used in pain management in the clinic. Therefore, in this study, we tested the hypothesis that morphine promotes the proliferation of cancer cell lines isolated from organs where opium increases cancer risk, namely, lung, bladder, and oesophagus, but not of cell lines isolated from organs where opium does not enhance cancer risk, namely, prostate, breast, or colon.

## 2. Results

**Figure 1:**
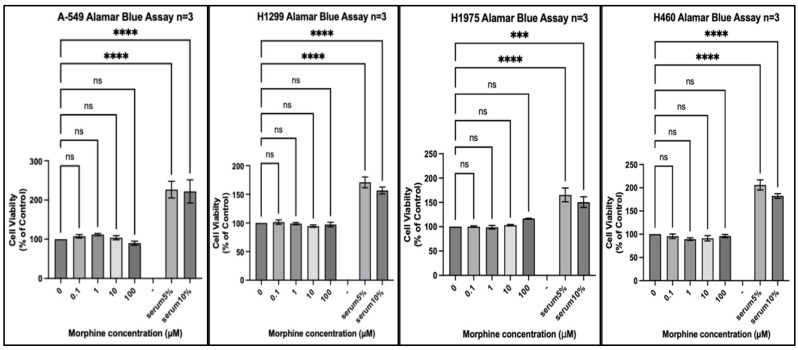
Proliferation of lung cancer cell lines (A-549, H1299, H1975, and H460) in response to morphine. Cells were exposed to indicated concentrations of morphine or serum for 48 h. Resazurin reduction was quantified after 4 h by fluorescence at an excitation of 560 nm and an emission of 590 nm. Results are expressed as a percentage of the viability of control cells unexposed to morphine or serum. Data are shown as mean ± SEM, n = 3 independent experiments. ns, not significant; *** *p* < 0.001; **** *p* < 0.0001.

**Figure 2:**
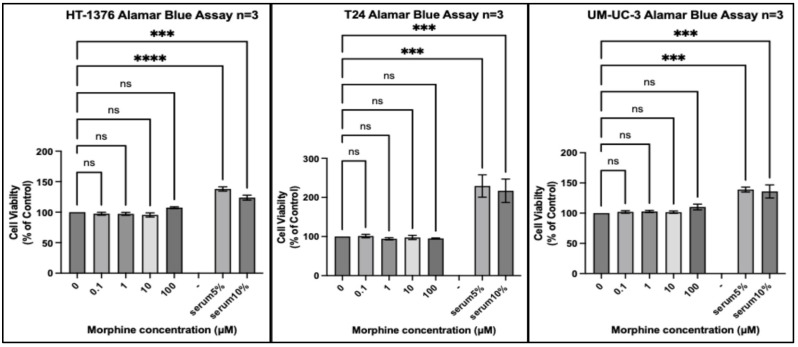
Proliferation of bladder cancer cell lines (HT-1376, T24, and UM-UC-3) in response to morphine. Cells were exposed to indicated concentrations of morphine or serum for 48 h. Resazurin reduction was quantified after 4 h by fluorescence at an excitation of 560 nm and an emission of 590 nm. Results are expressed as a percentage of the viability of control cells unexposed to morphine or serum. Data are shown as mean ± SEM, n = 3 independent experiments. ns, not significant; *** *p* < 0.001; **** *p* < 0.0001.

**Figure 3:**
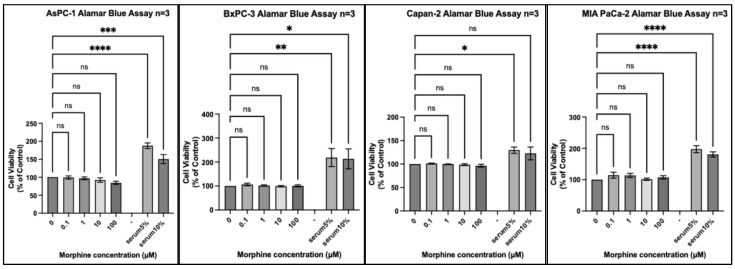
Proliferation of pancreas cancer cell lines (AsPC-1, BxPC-3, Capan-2, and MIA PaCa-2) in response to morphine. Cells were exposed to indicated concentrations of morphine or serum for 48 h. Resazurin reduction was quantified after 4 h by fluorescence at an excitation of 560 nm and an emission of 590 nm. Results are expressed as a percentage of the viability of control cells unexposed to morphine or serum. Data are shown as mean ± SEM, n = 3 independent experiments. ns, not significant; * *p* < 0.05; ** *p* < 0.01; *** *p* < 0.001; **** *p* < 0.0001.

**Figure 4:**
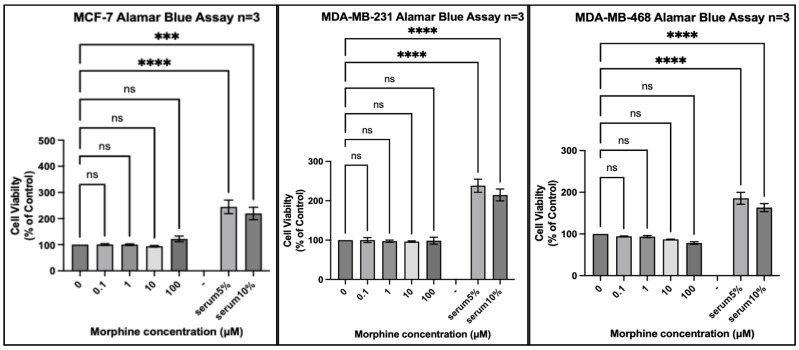
Proliferation of breast cancer cell lines (MCF-7, MDA-MB-231, and MDA-MB-468) in response to morphine. Cells were exposed to indicated concentrations of morphine or serum for 48 h. Resazurin reduction was quantified after 4 h by fluorescence at an excitation of 560 nm and an emission of 590 nm. Results are expressed as a percentage of the viability of control cells unexposed to morphine or serum. Data are shown as mean ± SEM, n = 3 independent experiments. ns, not significant; *** *p* < 0.001; **** *p* < 0.0001.

**Figure 5:**
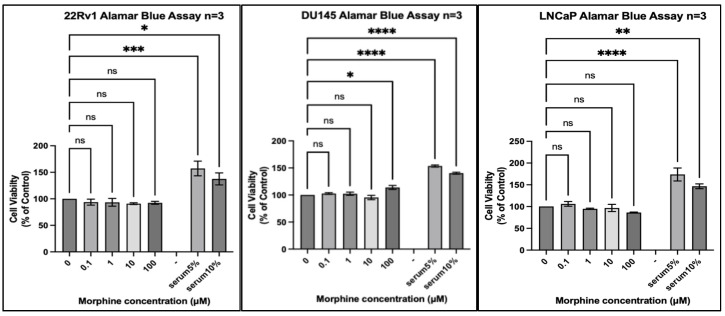
Proliferation of prostate cancer cell lines (22Rv1, DU145, and LNCaP) in response to morphine. Cells were exposed to indicated concentrations of morphine or serum for 48 h. Resazurin reduction was quantified after 4 h by fluorescence at an excitation of 560 nm and an emission of 590 nm. Results are expressed as a percentage of the viability of control cells unexposed to morphine or serum. Data are shown as mean ± SEM, n = 3 independent experiments. ns, not significant; * *p* < 0.05; ** *p* < 0.01; *** *p* < 0.001; **** *p* < 0.0001.

**Figure 6:**
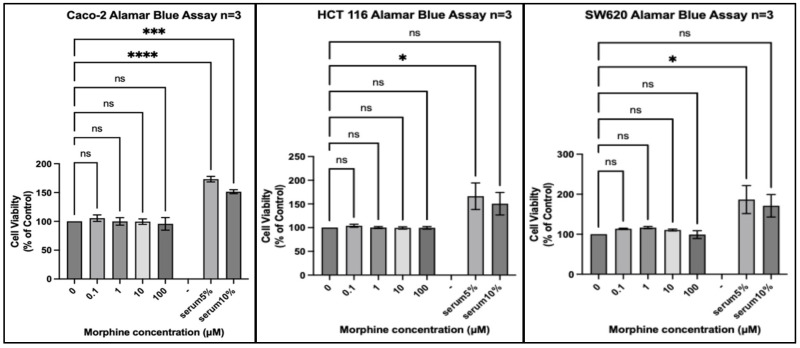
Proliferation of colon cancer cell lines (Caco-2, HCT 116, and SW620) in response to morphine. Cells were exposed to indicated concentrations of morphine or serum for 48 h. Resazurin reduction was quantified after 4 h by fluorescence at an excitation of 560 nm and an emission of 590 nm. Results are expressed as a percentage of the viability of control cells unexposed to morphine or serum. Data are shown as mean ± SEM, n = 3 independent experiments. ns, not significant; * *p* < 0.05; *** *p* < 0.001; **** *p* < 0.0001.

We tested 20 different cancer cell lines from six different organs: three organs reported by the IARC monograph to have strong evidence for increased risk of cancer associated with opium use and three organs either reported to not have, or lacking reports of, having an increased risk of cancer associated with opium use. Each experiment included a positive control of foetal bovine serum (FBS) 5% (*v*/*v*) and FBS 10% (*v*/*v*) to ensure that a potential lack of an effect of morphine did not result from the experimental conditions. In every experiment, all cell lines exposed to FBS 5% or FBS 10% for the same duration as morphine exhibited significantly increased proliferation (Figure 1, Figure 2, Figure 3, Figure 4, Figure 5 and Figure 6).

The first set of cell lines were isolated from the lung. The results (Figure 1) show no statistically significant change in proliferation across the four cell lines at any concentration of morphine. A slight biphasic trend was noted with the A-549 cell line, with a small increase at 1 μM and a decrease at 100 µM, both non statistically significant.

Similarly, in cells lines isolated from the bladder (Figure 2), no statistically significant change in proliferation across the three cell lines was observed at any concentration of morphine, despite a slight but non-significant increase with 100 µM morphine for the HT-1376 cells.

The same results were obtained with cell lines isolated from the pancreas (Figure 3), where no statistically significant change in proliferation across the four cell lines was observed at any concentration of morphine. There was a trend towards decreased proliferation with 100 µM morphine in the AsPC-1 cell line, which was not statistically significant.

Comparable results were observed in cancer cell lines isolated from the breast (Figure 4), with no statistically significant change in proliferation observed across the three cell lines at any concentration of morphine. There was a trend of increased proliferation in the MCF-7 cell line at morphine 100 µM, and decreased proliferation observed in MDA-MB-468 at 100 µM.

The same results were observed in cell lines isolated from the prostate (Figure 5), where only DU145 exhibited a statistically significant increased proliferation at 100 μM morphine, but no other statistically significant change was observed across the three cell lines. A slight decrease was seen in LNCaP at 100 µM morphine; however, this was not statistically significant.

Finally, in cell lines isolated from the colon (Figure 6), a similar trend was seen, with no statistically significant change in proliferation observed across the three cell lines. A biphasic effect was seen in SW620 with a small increase at morphine 1 µM and a small decrease seen at morphine 100 µM; however, this was not statistically significant.

**Figure A1:**
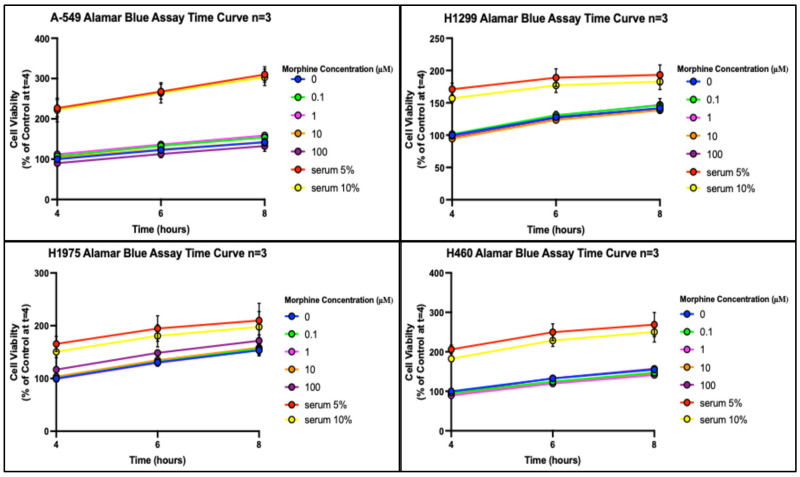
Proliferation of lung cancer cell lines (A-549, H1299, H1975, and H460) in response to morphine. Cells were exposed to indicated concentrations of morphine or serum for 48 h. Resazurin reduction was quantified after 4, 6, and 8 h by fluorescence at an excitation of 560 nm and an emission of 590 nm. Results are expressed as a percentage of the viability of control cells unexposed to morphine or serum at time = 4 h. Data are shown as mean ± SEM, n = 3 independent experiments.

**Figure A2:**
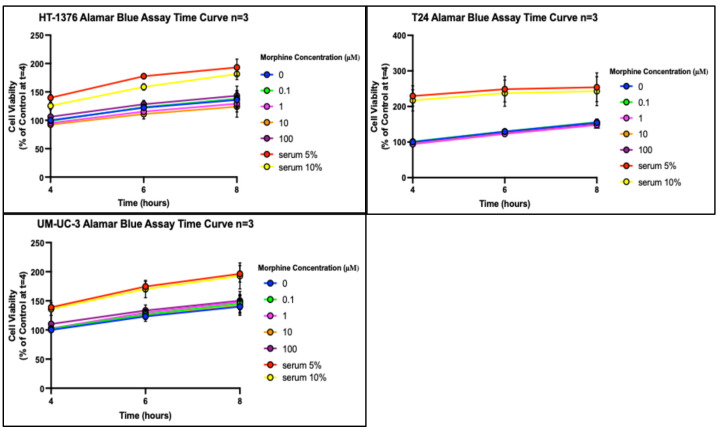
Proliferation of bladder cancer cell lines (HT-3376, T24, and UM-UC-3) in response to morphine. Cells were exposed to indicated concentrations of morphine or serum for 48 h. Resazurin reduction was quantified after 4, 6, and 8 h by fluorescence at an excitation of 560 nm and an emission of 590 nm. Results are expressed as a percentage of the viability of control cells unexposed to morphine or serum at time = 4 h. Data are shown as mean ± SEM, n = 3 independent experiments.

**Figure A3:**
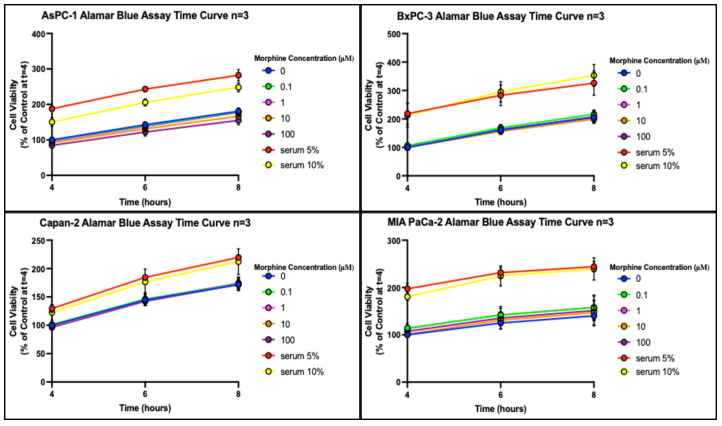
Proliferation of pancreas cancer cell lines (AsPC-1, BxPC-3, Capan-2, and MIA PaCa-2) in response to morphine. Cells were exposed to indicated concentrations of morphine or serum for 48 h. Resazurin reduction was quantified after 4, 6, and 8 h by fluorescence at an excitation of 560 nm and an emission of 590 nm. Results are expressed as a percentage of the viability of control cells unexposed to morphine or serum at time = 4 h. Data are shown as mean ± SEM, n = 3 independent experiments.

**Figure A4:**
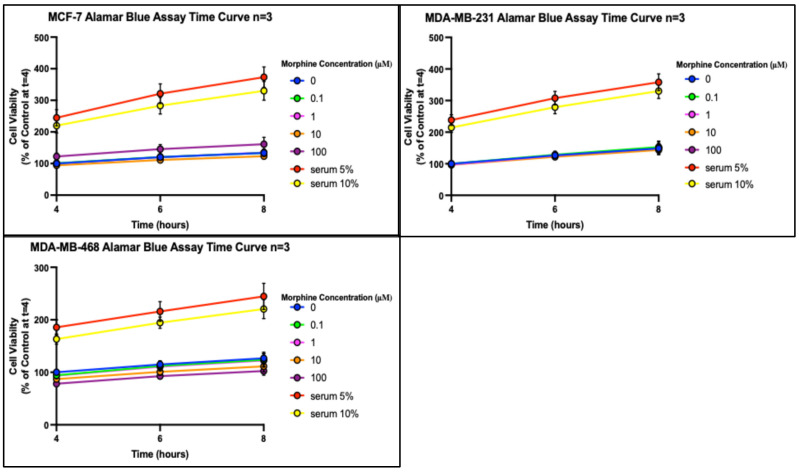
Proliferation of breast cancer cell lines (MCF-7, MDA-MB-231, and MDA-MB-468) in response to morphine. Cells were exposed to indicated concentrations of morphine or serum for 48 h. Resazurin reduction was quantified after 4, 6, and 8 h by fluorescence at an excitation of 560 nm and an emission of 590 nm. Results are expressed as a percentage of the viability of control cells unexposed to morphine or serum at time = 4 h. Data are shown as mean ± SEM, n = 3 independent experiments.

**Figure A5:**
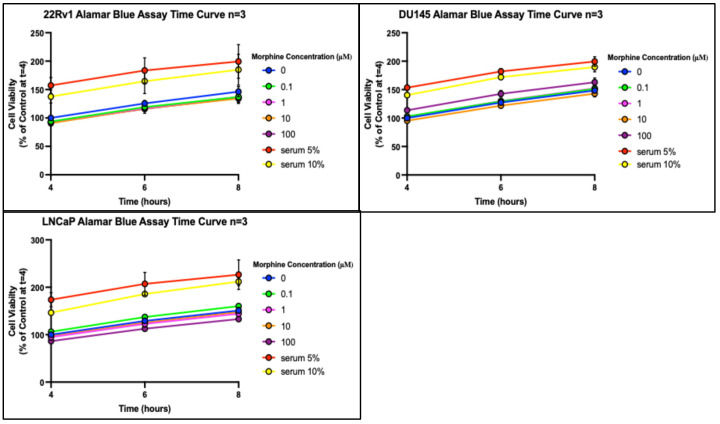
Proliferation of prostate cancer cell lines (22Rv1, DU145, and LNCaP) in response to morphine. Cells were exposed to indicated concentrations of morphine or serum for 48 h. Resazurin reduction was quantified after 4, 6, and 8 h by fluorescence at an excitation of 560 nm and an emission of 590 nm. Results are expressed as a percentage of the viability of control cells unexposed to morphine or serum at time = 4 h. Data are shown as mean ± SEM, n = 3 independent experiments.

**Figure A6:**
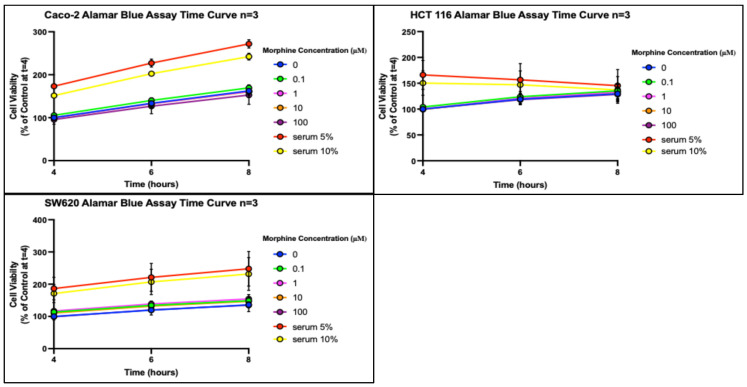
Proliferation of colon cancer cell lines (Caco-2, HCT 116, and SW620) in response to morphine. Cells were exposed to indicated concentrations of morphine or serum for 48 h. Resazurin reduction was quantified after 4, 6, and 8 h by fluorescence at an excitation of 560 nm and an emission of 590 nm. Results are expressed as a percentage of the viability of control cells unexposed to morphine or serum at time = 4 h. Data are shown as mean ± SEM, n = 3 independent experiments.

We also measured cell viability at 6 and 8 h and expressed viability as a percentage of the negative control at t = 4 h to generate time course curves. The results are available in Appendix A (Figure A1, Figure A2, Figure A3, Figure A4, Figure A5 and Figure A6). They display the same trend seen at the 4 h timepoint but show that cell viability increased with time across all cell lines at all concentrations, further confirming the appropriateness of our experimental setting.

## 3. Discussion

The recent listing of opium consumption as carcinogenic to humans for only certain organs by the IARC monograph raises the question of how each body site can play a role in determining the growth of a tumour in response to opioids. With opioid alkaloids such as morphine, which is commonly used in chronic pain management, it remains relevant to test the relationship between morphine and explore the difference between cancer cells isolated from various organs. We evaluated three to four cancer cell lines each from three organs that, as per the IARC monograph, have an increased risk of cancer associated with opium use, and three to four cell lines each from three body sites that are either reported to not have, or are lacking reports of, an increased risk of cancer associated with opioid use, to gain insight into this potential relationship.

Our data indicate that if morphine has an effect on tumour growth, then it is not through a direct effect on the proliferation of the cancer cells. Our results consistently show a lack of effect of morphine on cancer cell proliferation in vitro at the concentrations and times that we tested and reveal no organ-specific variation. Small-scale increases or decreases in proliferation, which were not statistically significant and of a magnitude questioning their biological significance, were seen at the highest concentration we used. Importantly, these effects are not consistent across organs, lending to the conclusion that morphine’s effect on cancer cell proliferation in vitro is not dependent on the organ of origin of the cancer cells.

We chose the range of concentrations used in our study based on clinical relevance. The fact that a trend towards either increased or decreased proliferation was seen at 100 µM may indicate that a bigger-sized effect of statistical significance would have been obtained with doses beyond 100 μM. However, concentrations found in the circulation of patients receiving morphine are much lower than what is often used in vitro. For example, in a study where patients’ morphine plasma levels were measured after morphine administration, pain relief was achieved at a plasma level of 40 ng/mL, which translates to 0.104 μM, with the highest level measured in this patient cohort using their regular clinical doses of 82 ng/mL, which translates to only 0.21 μM [29]. More recent studies report that higher therapeutic doses of 10–2400 mg/day can result in serum concentrations of 2–3.5 μM [30]. Even advanced cancer patients who require higher morphine doses have concentrations recorded of up to 1440 ng/mL which translates to 5.05 μM [31]. Therefore, our choice of concentrations was well suited to encompass all possible exposure that may occur in cancer cells in the clinical situation. This is in contrast to some in vitro studies that reach up to 10 mM [14,32].

There are over 230 published studies using various opioids that assess proliferation of cancer cells in vitro that show increased [33,34], decreased [35,36], or no change [8,37] in proliferation when using opioids on various cancer cell lines from multiple organs. It is difficult to reconcile and compare results due to the wide range of opioid molecules employed, concentrations ranging from 0.1 nM to 10 mM, duration of treatment varying from 120 min to up to 28 days, different protocols (e.g., starving or not starving the cells before opioid treatment), and different tests measuring viability and proliferation (e.g., 3-(4,5-dimethylthiazolyl-2)-2,5-diphenyltetrazolium bromide (MTT) assay, colony formation, etc.). For example, two studies that investigated A-549 proliferation in response to morphine used different concentrations, different seeding protocols, different timepoints, and different assays, and one found that there was an increase in proliferation, while the other observed a decrease [13,24]. Our study presents the advantage of testing all cell lines with an unchanged protocol between cell lines after extensive preliminary work to ensure its application was repeatable, while keeping all treatment conditions the same except for the culture medium the cells are grown in, which respects what their maintenance requires.

We also recorded the cell viability at 6 and 8 h and compared this to the negative control at t = 4 h, which is often overlooked, resulting in lack of data on a time course of proliferation or death in other studies. We observed continued cell growth in all cell lines across both the positive and negative controls and in cells treated with morphine. This indicates that our experimental conditions were appropriately chosen to detect an effect of morphine.

**Figure A7:**
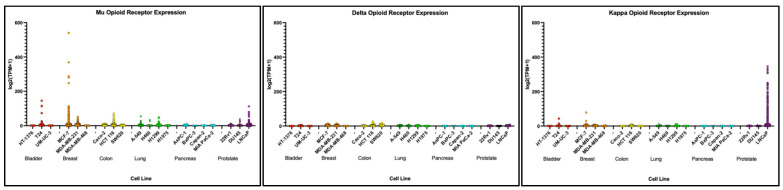
Gene expression of mu (**left**), delta (**centre**), and kappa (**right**) opioid receptors expressed in transcripts per million (TPM), taken from the PCTA.

One potential explanation for an organ-specific effect of opioids on proliferation of cancer cells could be a difference in expression of opioid receptors by the epithelial/cancer cells in different organs. Morphine has the greatest affinity for MOR, with lesser affinity for kappa (KOR) and delta opioid receptors (DOR). We extracted the RNA expression of the mu, delta, and kappa opioid receptors of the cell lines used in our study (Appendix B, Figure A7) from a resource centralising the existing data reporting gene expression of these receptors in cancer cell lines, the Pan-Cancer Cell Line Transcriptome Atlas (PCTA) [38]. The data indicate that expression of DOR and KOR is extremely low across the majority of cell lines used in our study, with only LNCaP having high KOR expression. Furthermore, MOR expression is low across most cells lines, and higher levels are not consistently attributed to a specific organ. The cell lines that do have higher MOR expression in PCTA, such as MCF-7 and T24, do not in our study exhibit proliferation changes upon exposure to morphine. This supports the conclusion that if there is an effect of morphine on cancer cell proliferation, it is not via a direct effect on the cancer cells mediated by opioid receptors. This aligns with studies showing that naloxone did not reverse the proliferative or anti-proliferative effect of morphine on cancer cells [22,39].

This work has significant implications on future directions of this research. The IARC monograph shows an organ-specific cancer risk increase in humans in relation to opium consumption, and emerging evidence indicates a similar effect for pharmaceutical opioids [26,27], though the underlying mechanism remains unknown. While it may still be the cancer cells themselves that are the target of this effect, it may not be simple proliferation upon morphine agonism. Tumour biology is complex, and the interactions between cancer cells and other actors of the tumour microenvironment (TME) play a major role in tumour growth and metastasis [40,41]. This complex relationship may be affected by opioids in a body site-specific fashion [42]. Other mechanisms that may mediate an organ-specific effect of opioids include the immune response. In favour of this hypothesis, the organ specificity of the TME immune landscape is increasingly scrutinised [43], and opioids are recognised to affect multiple aspects of the anticancer immune response [44], but to date it is unknown whether morphine affects the TME differently in various body sites. Angiogenesis is another proposed indirect mechanism for organ specificity. Novel tumour vasculature derives primarily from a preexisting microvascular endothelium, which differs significantly between organs [45,46]. It is conceivable that expression of pro-angiogenic or anti-angiogenic factors differs in the preexisting vasculature between organs [47,48,49], and that overexpression of these factors varies between tumours developing in different body sites. For instance, the epidermal growth factor receptor that promotes angiogenesis and activation of EGFR signalling is known to enable an intravasation-sustaining microenvironment in the developing primary tumour [50]. This effect may mediate organ specificity of opioids, as it has been shown that EGFR mutations are most frequent in lung adenocarcinomas and least frequent in breast carcinomas [51]. There is evidence that the mu opioid receptor regulates opioid and growth factor-induced EGF receptor signalling [52].

Our study is limited to one of the many mechanisms by which opioids may influence tumour biology, namely, cancer cell proliferation. Our results do not preclude the other potential effects of morphine on cancer cell migration, metastasis and angiogenesis, or the indirect effects of morphine—for example, via immunosuppression. Our results are also limited by the range of concentrations of morphine used, as the study may have benefited from using concentrations much higher than clinically relevant. Using morphine, the most abundant glucuronides, such as M3G and M6G, would also have improved translation of our work. However, our study presents several strengths: (i) Our results between cells lines and organs are directly comparable due to our experimental design, (ii) we starved the cells for 24 h to maximise the chances of unveiling a stimulation of proliferation, and (iii) we observed increased proliferation with a serum-positive control included in each experiment to demonstrate that the experimental conditions were favourable for cell growth.

## 4. Conclusions

Given the recent classification of opium consumption as carcinogenic to humans by the IARC monograph, and the emerging evidence that shows the increase in cancer risk in the same opium-related organs in relation to using pharmaceutical opioids [26,27], mechanistic studies exploring mechanisms that may underlie the observed effects in epidemiological data are needed. We show a lack of effect for morphine on the proliferation of twenty different human cancer cell lines encompassing six different organs in our experimental conditions and reveal no variation that was organ-specific. Future research focus should explore how indirect mechanisms, such as the immune response, angiogenesis, and varying elements of the TME, may affect tumour growth in an organ-specific fashion.

## 5. Materials and Methods

### 5.1. Materials

Cell culture medium, serum, and supplements were from Life Technologies (Mulgrave, VIC, Australia). Morphine sulphate was purchased from Hospira (Mulgrave, VIC, Australia). Other reagents, including resazurin sodium salt (R7017), were purchased from Sigma-Aldrich (Castle Hill, NSW, Australia).

### 5.2. Cell Maintenance

All cells were kept at 37 °C in a humidified atmosphere comprising 5% CO_2_ and passaged when 90% confluent. The following cells (followed by their ATCC catalogue number) were cultured in DMEM supplemented with foetal bovine serum (FBS) 10% (*v*/*v*), 100 U/mL penicillin, and 100 μg/mL streptomycin: lung adenocarcinoma A-549 (CRM-CCL-185), invasive breast carcinoma MCF-7 (HTB-22), breast adenocarcinoma MDA-MB-231 (CRM-HTB-26), breast adenocarcinoma MDA-MB-468 (HTB-132), pancreatic ductal adenocarcinoma MIA PaCa-2 (CRM-CRL-1420), colon adenocarcinoma SW620 (CCL-227), bladder carcinoma UM-UC-3 (CRL-1749) (with 2 mM l-glutamine, 0.1 mM non-essential amino acids, and 1 mM sodium pyruvate added), and bladder carcinoma HT-1376 (CRL-1472) (with 2 mM l-glutamine, 0.1 mM non-essential amino acids, and 1 mM sodium pyruvate added). The following cells were cultured in RPMI medium supplemented with foetal bovine serum (FBS) 10% (*v*/*v*), 100 U/mL penicillin, and 100 μg/mL streptomycin: lung large cell carcinoma H1299 (CRL-5803), lung adenocarcinoma H1975 (CRL-5908), lung large cell carcinoma H460 (HTB-177), pancreatic ductal adenocarcinoma AsPC-1 (CRL-1682), pancreatic ductal adenocarcinoma BxPC-3 (CRL-1687), prostate carcinoma 22Rv1 (CRL-2505), prostate carcinoma DU145 (HTB-81), prostate carcinoma LNCaP (CRL-1740), and colon adenocarcinoma Caco-2 (HTB-37) (with 0.1 mM non-essential amino acids added). The following cells were cultured in McCoys 5A (modified) medium supplemented with foetal bovine serum (FBS) 10% (*v*/*v*), 100 U/mL penicillin, and 100 μg/mL streptomycin: pancreatic ductal adenocarcinoma Capan-2 (HTB-80), colon carcinoma HCT 116 (CCL-247), and bladder carcinoma T24 (HTB-4) (with 2 mM l-glutamine, 0.1 mM non-essential amino acids, and 1 mM sodium pyruvate added).

### 5.3. Cell Proliferation Assay

Cells were seeded into black wall–clear base 96 well plates at 3000 cells per well in each cell’s respective medium with serum and incubated for 24 h to allow for cell adherence. The medium was then replaced with 100 μL of the cells’ respective medium with no serum and incubated for 24 h. The medium was then replaced with 100 μL of each experimental condition in quintuplicate. Exposure to morphine occurred in no serum medium: 0 μM morphine, 0.1 μM morphine, 1 μM morphine, 10 μM morphine, and 100 μM morphine. Proliferation positive controls were 5% (*v*/*v*) foetal bovine serum and 10% (*v*/*v*) foetal bovine serum. Plates were incubated for 48 h. The medium was then aspirated from the wells, and the cells were rinsed with 100 μL of phosphate buffer solution (PBS). The cells were placed in 100 μL of resazurin 1 μM in their respective serum-free medium and incubated at 37 °C. The resazurin was taken up by viable cells and reduced within their cytosol into a product that fluoresces at 590 nm and is released into the medium. Fluorescence was read after 4, 6, and 8 h with excitation at 560 nm and emission at 590 nm, a measurement height of 9.5 mm, and 100 flashes in an EnSight multimode plate reader (PerkinElmer). Data were processed in Excel and expressed as a percentage of the viability of negative control cells, then graphed using Prism software version 10. Statistical analysis was conducted using ordinary one-way ANOVA with Dunnett’s post-test and compared to the negative control. Statistical significance was recognised at *p* < 0.05.

## Data Availability

The original contributions presented in the study are included in the article/Appendix A and Appendix B, further inquiries can be directed to the corresponding authors.
